# Gene Ontology annotation highlights shared and divergent pathogenic strategies of type III effector proteins deployed by the plant pathogen *Pseudomonas syringae *pv *tomato *DC3000 and animal pathogenic *Escherichia coli *strains

**DOI:** 10.1186/1471-2180-9-S1-S4

**Published:** 2009-02-19

**Authors:** Magdalen Lindeberg, Bryan S Biehl, Jeremy D Glasner, Nicole T Perna, Alan Collmer, Candace W Collmer

**Affiliations:** 1 Department of Plant Pathology, Cornell University, Ithaca, NY 14850, USA; 2 Department of Genetics, University of Wisconsin, Madison WI, USA; 3 Department of Biological and Chemical Sciences, Wells College, Aurora, NY 13026, USA

## Abstract

Genome-informed identification and characterization of Type III effector repertoires in various bacterial strains and species is revealing important insights into the critical roles that these proteins play in the pathogenic strategies of diverse bacteria. However, non-systematic discipline-specific approaches to their annotation impede analysis of the accumulating wealth of data and inhibit easy communication of findings among researchers working on different experimental systems. The development of Gene Ontology (GO) terms to capture biological processes occurring during the interaction between organisms creates a common language that facilitates cross-genome analyses. The application of these terms to annotate type III effector genes in different bacterial species – the plant pathogen *Pseudomonas syringae *pv *tomato *DC3000 and animal pathogenic strains of *Escherichia coli *– illustrates how GO can effectively describe fundamental similarities and differences among different gene products deployed as part of diverse pathogenic strategies. In depth descriptions of the GO annotations for *P. syringae *pv *tomato *DC3000 effector AvrPtoB and the *E. coli *effector Tir are described, with special emphasis given to GO capability for capturing information about interacting proteins and taxa. GO-highlighted similarities in biological process and molecular function for effectors from additional pathosystems are also discussed.

## Background

Bacterial pathogens subvert defenses and generate favorable niches in diverse eukaryotes through an array of extracellular factors. Most of these factors are proteins transported out of the bacterial cell by one of several secretion pathways, numerically distinguished as type I through type VI. Proteins secreted by the type III secretion system (T3SS) are critical to pathogens of diverse hosts; the best studied of these bacteria include enteric pathogens of animals, *E. coli*, *Salmonella*, and *Yersinia*, and plant pathogens in the genera *Pseudomonas*, *Xanthomonas*, and *Ralstonia *[1,2]. Related secretion systems are utilized during beneficial types of symbiosis, with the best studied being the T3SS in the nitrogen-fixing legume symbionts, *Rhizobium *[3,4].

The ever-growing number of complete genome sequences has prompted intensive genome-informed investigations of Type III effector repertoires in various bacterial strains and species. However, a long history of non-systematic, discipline-specific approaches to the annotation of Type III effectors (and virulence genes in general) has created a significant impediment to rapid analysis of this wealth of new data. Effector gene names typically vary, with two or more three-letter gene names often used for effectors from the same species (e.g., *sip *and *sop *in *Salmonella *or *avr *and *hop *in *P. syringae*) and annotation of product names being similarly idiosyncratic. Attempts have been made to systematize three letter gene name assignments for select groups of organisms [5,6], but even with a rigorously applied nomenclature, only limited information can be captured in this way. Furthermore, the high rates of horizontal transfer observed for effector genes [5], coupled with an accelerated rate of evolution for some [7,8], can confound recognition of related effectors through standard analysis of homology. As a result, researchers interested in broad comparisons of gene products and pathosystems must invest significant amounts of time piecing together their own summary from the primary literature.

## The Gene Ontology – a universal language for capturing effector characteristics

The Gene Ontology (GO) was originally developed by researchers working on eukaryotic model systems as a controlled vocabulary for describing processes common to diverse organisms [9]. The three ontologies that make up GO are designed to capture information on the cellular location, molecular functions, and biological processes that characterize individual gene products. GO terms are organized into a tree-like hierarchy where more general, high level terms are the parents of more specific child terms. Gene products can be annotated to as many terms as are applicable, with the level of specificity dependent on the extent of characterization. Evidence codes attached to individual annotations document the nature of supporting evidence and identifiers for the associated publications provide direct links to the source of the data.

The Plant-associated Microbe Gene Ontology (PAMGO) project  was initiated for the purpose of creating GO terms that specifically capture cellular locations and biological processes relevant to interactions between organisms. Of the more than 700 new GO terms created as part of this project; most are found under the "interspecies interaction between organisms" parent in the Biological Process Ontology. Term development has been accompanied by focused efforts on the part of PAMGO members to comprehensively annotate effectors in selected bacterial pathogens – specifically, the plant pathogen *Pseudomonas syringae *pv *tomato *DC3000 (*Pto *DC3000) and numerous enterics including the plant pathogen *Dickeya dadantii *and animal pathogenic strains of *E. coli*.

*Pto *DC3000 and *E. coli *0157:H7 represent useful case studies for initiation of a global effector annotation project. Both pathogens require a wide range of T3SS-dependent effectors to establish infection within their respective hosts. Furthermore, as pathogens of hosts in both the plant and animal kingdoms, they illustrate the utility of GO's multi-level structure for conceptualizing shared and divergent aspects of their pathogenic strategies.

## *Pseudomonas syringae pv. tomato *DC3000

*Pto *DC3000 is a pathogen of tomato and *Arabidopsis*, was the first *P. syringae *strain sequenced to completion, and is a model for the study of bacterial-plant interactions [10]. T3SS effector proteins, identified on the basis of their regulation by the HrpL alternative sigma factor and their passage out of the bacterial cell via the T3SS, have long been known to play a critical role in pathogenicity and host-range determination of *P. syringae *pathovars. Indeed, cataloguing their complete repertoire represented one of the chief motivations for sequencing the *Pto *DC3000 genome. More than 50 effector families, defined by phylogenetic grouping [11], have been identified among the *P. syringae *pathovars, with over 36 families found in *Pto *DC3000. The majority of these were identified using a combination of BLAST analysis of predicted genes against previously identified effectors and iterative pattern-based searches using the conserved HrpL binding site and N-terminal sequence patterns associated with T3SS targeting [11].

**Figure 1:**
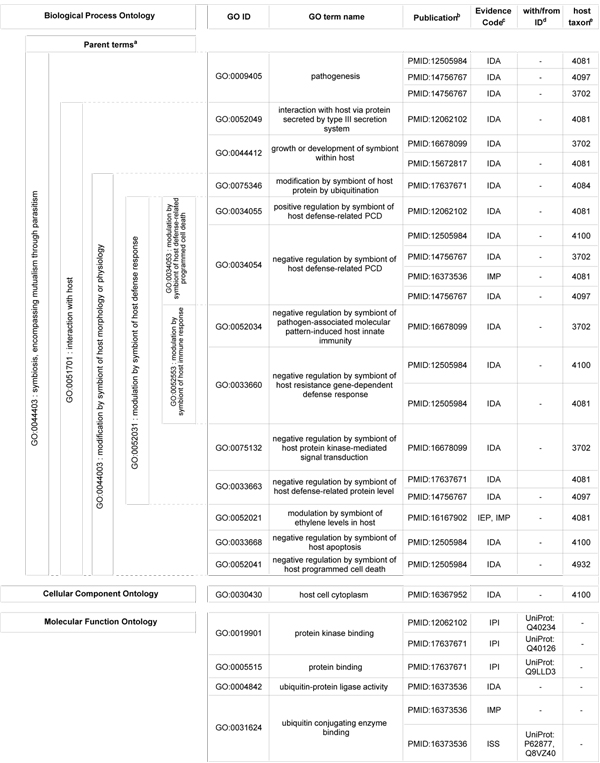
**Gene Ontology annotation for the Pto DC3000 Type III effector AvrPtoB**. ^a^Indicates the nearest common parent term in the GO term hierarchy. Terms sharing the specified parent are delimited by dashed lines. ^b^Indicates the publication supporting annotation of AvrPtoB to the specified GO term. ^c^Indicates the nature of the evidence supporting the annotation; IDA, inferred from direct evidence; IEP, inferred from expression profile; IPI, inferred from physical interaction; IMP, inferred from mutant phenotype; ISS, inferred from sequence similarity. ^d^Indicates the Uniprot accession number of the interacting protein, where inferred from physical evidence, or of the similar protein, where inferred from sequence similarity. ^e^Indicates the taxon ID of the host where biological processes occurred in relation to a host organism.

Since their initial identification as substrates of the T3SS, research on the *Pto *DC3000 effectors has yielded new insights into their molecular functions, cellular destinations within the host, and the biological processes in which they participate. To date, over 300 Gene Ontology annotations have been generated for 36 effector genes as part of the PAMGO project, with the vast majority of annotations concerning processes that occur during the interaction between microbes and their host organisms. An example of the range of processes in which the effectors engage is illustrated by the *Pto *DC3000 effector protein AvrPtoB (HopAB2) (see the table in Figure 1). Like many other effectors, AvrPtoB is annotated to terms in all three ontologies. Within the Biological Process Ontology, terms range from the more general such as "GO:0009405 pathogenesis" and "GO:0044412 growth or development of symbiont within the host", applicable to a wide range of virulence factors in diverse pathogens, to more specific terms such as "GO:0052049 interaction with host via protein secreted by type III secretion system" that specifically identifies the Type III effectors.

AvrPtoB and other *P. syringae *effectors play significant roles in modulating the host defense response (GO:0052031), and as part of PAMGO term development, an extensive tree of child terms was created to capture the variety of processes contributing to this phenomenon. Though many of these terms are, at least for the present, used only for bacteria-plant interactions, it is critical to the utility of GO that terms be defined using language that is meaningful across many pathosystems. The phrase "hypersensitive response" serves as a useful example. While this term is commonly used among plant pathologists to refer to rapid defense-associated plant cell death at the site of infection, to researchers in animal systems it can have very different allergy-related or behavioral connotations. Therefore, newly developed PAMGO terms avoid using "hypersensitive response" in the term name and instead use term names such as "GO:0034053 modulation by symbiont of host defense-related programmed cell death" to annotate such bacterial effector activity. At the same time, the previously existing GO term "GO:0009626 hypersensitive response" was modified at the request of PAMGO collaborators to "plant-type hypersensitive response," thus clearly matching the new term name with the existing GO definition, which specified plant cells. It is important to note that an investigator searching GO terms for "hypersensitive response" would be pointed to both terms named above by means of the synonym field attached to each GO term. Finally, these two terms illustrate the difference between terms appropriate for annotating genes in the microbe, or symbiont (GO:0034053) versus those in the plant (GO:0009626) that are involved in the process described.

AvrPtoB is annotated to several child terms of "GO:0052031 modulation by symbiont of host defense response" including: "GO:0034054 negative regulation by symbiont of host defense-related programmed cell death [PCD]", "GO:0034055 positive regulation by symbiont of host defense-related programmed cell death", "GO:0033660 negative regulation by symbiont of host resistance gene-dependent defense response", "GO:0075132 negative regulation by symbiont of host protein kinase-mediated signal transduction", and "GO:0052034 negative regulation by symbiont of pathogen-associated molecular pattern-induced host innate immunity". At first glance, these annotations may appear contradictory – after all, how can the same gene product be annotated to both "GO:0034055 positive regulation by symbiont of host defense-related PCD" and "GO:0034054 negative regulation by symbiont of host defense-related PCD"? In this case, the answer lies in the secondary or dual taxon field incorporated into the GO database as part of the PAMGO project. This field functions to indicate the identities of the organisms between which the interaction is occurring. Thus, closer examination reveals that "GO:0034055 positive regulation by symbiont of host defense-related PCD" applies to AvrPtoB in the *Pto *DC3000 interaction with *S. lycopersicum *(tomato) while "GO:0034054 negative regulation by symbiont of host defense-related PCD" is used to annotate the interaction between *Pto *DC3000 and *Nicotiana benthamiana *(tobacco). In fact, annotation to "GO:0034054 negative regulation by symbiont of host defense-related PCD" is shown in triplicate to reflect interactions of Pto DC3000 in three separate hosts – *Nicotiana benthamiana*, *Nicotiana tabacum *cv. *Xanthi*, and *Arabidopsis thaliana*. Where additional clarification of strains and genotypes of interacting organisms is required, users can refer to the associated publications found in the reference field of the GO annotation.

In addition to annotations in the Biological Process ontology, annotations to the Cellular Component and Molecular Function ontologies are also shown. As one of the most thoroughly characterized of the *Pto *DC3000 effectors, AvrPtoB has several Molecular Function annotations that provide insight on the specific enzymatic and binding capabilities by which AvrPtoB accomplishes the processes described above. Molecular Function annotations include: "GO:0019901 protein kinase binding", "GO:0004842 ubiquitin-protein ligase activity", and "GO:0031624 ubiquitin conjugating enzyme binding". Just as documenting the taxa of interacting organisms is critical to the usefulness of biological process terms, so documentation of interacting proteins significantly enhances the value of Molecular Function terms. For example, the interacting proteins for "GO:0019901 protein kinase binding" are Q40234 (Pto) and Q40126 (Fen), and for "GO:0005515 protein binding", Q9LLD3 (PtoC). Most of the evidence codes used for AvrPtoB indicate experimental evidence for the assigned annotations, including IDA (inferred from direct assay), IMP (inferred from mutant phenotype), and IPI (inferred from physical interaction). In contrast, the evidence code ISS (inferred from sequence or structural similarity) indicates that the annotation is based on similarity of the given gene product to an experimentally characterized homolog. Annotations made on the basis of sequence or structural similarity require that the ID of the protein from which the annotation is inferred be included in the with/from column. Unlike AvrPtoB, for which the ISS code is used only once to capture its structural similarity to known E3 ubiquitin ligases (UniProt: P62877, Q8VZ40), GO annotations for effectors in some other *P. syringae *strains rely more extensively on sequence similarity. In such cases where experimental evidence is lacking, sequence similarity to Pto DC3000 effectors can be used to guide GO annotation of those effectors. (Some important considerations relevant to propagating GO annotations based on sequence similarity are described in the following section.) When sequence similarity is absent, GO annotations can provide clues to candidate functions or biological processes in newly identified gene products based on annotations previously made for other experimentally characterized gene products. For example, once a newly described gene product is found to be secreted and thus annotated to "GO:0052049 interaction with host via protein secreted by type III secretion system", other processes associated with this annotation in other experimentally characterized effectors become candidates for testing. These might include "GO:0044412 growth or development of symbiont within host", "GO:0034055 positive regulation by symbiont of host defense-related PCD", or "GO:0052034 negative regulation by symbiont of pathogen-associated molecular pattern-induced host innate immunity".

## *Escherichia coli*

Like *P. syringae*, many strains of *E. coli *rely on effectors to establish a pathogenic relationship with their host and are the focus of intense interest owing to their ability to cause serious disease in humans. Numerous genomes have recently been sequenced from pathogenic and non-pathogenic *E. coli *strains, and no one strain serves as a general model for the diverse pathogenic strategies found within this species. Consequently, PAMGO consortium members working on the Enterobacteriaceae, in contrast to those working on *P. syringae*, have focused on automated propagation of annotations from a handful of experimentally characterized effectors to homologs in numerous complete and draft genomes of *E. coli *and other enteric bacteria.

*E. coli *O157:H7 provides a representative example of the current state of experimentally-based annotation of enteric effectors and also reveals the limitations of transitive or similarity-based propagation of annotations. A total of 39 type III effectors have been identified in *E. coli *0157:H7 strain Sakai through a combination of bioinformatics, "secretome" analysis, and translocation assays with Cya fusions [5]. However, the absence of a convenient model host system for *E. coli *O157:H7 has impeded *in vivo *characterization of host interaction phenotypes on the level conducted in *P. syringae*, with the result that annotation of *E. coli *O157:H7 effectors has relied more extensively on inferences made from sequence similarity.

Among the O157:H7 effectors studied in greater depth is the Translocated Intimin Receptor protein (Tir), which plays a key role in bacterial attachment to host cells [12-15]. Given that attachment has proven a tractable process for studying in cell culture models, it is possible to assign GO annotations to Tir with a specificity comparable to that of AvrPtoB. Tir is secreted into host cells via the LEE (locus of enterocyte effacement) T3SS and then trafficked to the host cell plasma membrane (GO:0020002), where it binds the (also LEE-encoded) intimin protein on the bacterial cell surface. This binding activity is captured by the combination of Molecular Function ontology terms "GO:0051635 bacterial cell surface binding" and "GO:0005515 protein binding" using the IPI evidence code and "with" qualifier to specify the interacting partner as intimin. The role of Tir in bacterial attachment is reflected by the Biological Process term "GO:0044406 adhesion to host", with subsequent effects of Tir on the cascade of host signaling, described by "GO:0052027 modulation by symbiont of defense-related host MAP kinase-mediated signal transduction pathway" and major host cytoskeletal remodeling captured by "GO:0052039 modification by symbiont of host cytoskeleton" easily accommodated by child terms of "GO:0051701 interaction with host". The term "GO:0052057 modification by symbiont of host morphology or physiology via protein secreted by type III secretion system" links Tir to other T3SS effectors, while "GO:0009405 pathogenesis" indicates its role in disease.

The Tir effector also highlights some of the challenges inherent to cross-genome term assignments. Given that transitive annotation based on sequence and structural similarity forms the basis of most annotations in the GO database, discussion of the limitations of such annotations is warranted. Specifically, similar gene products involved in the interaction between organisms can have very different properties depending on both their source organism and the host with which they are interacting. For example, Tir has been shown to have different molecular functions depending on whether it is produced by enterohemorrhagic (EHEC) O157:H7 or enteropathogenic (EPEC) strains of *E. coli*. In EPEC, but not EHEC, tyrosine phosphorylation of Tir by the bacterium plays a key role in initiating the host cell signaling cascades and the cytoskeletal rearrangements involved with formation of a pedestal-like structure immediately under the adherent bacterial cell [13,14]. It is therefore imperative that the annotations assigned to EPEC-derived Tir not be propagated to Tir from EHEC strains. GO provides the option of using the qualifier "NOT" together with an annotation such as "GO:0019901 protein kinase binding" to indicate that the *E. coli *O157:H7 Tir protein is not phosphorylated. However, many GO annotation repositories, including the UW ASAP database of enterobacterial genomes [16]), do not display this qualifier by default, with the result that the "NOT" qualifier is used infrequently. A more in depth discussion of the "NOT" qualifier and differences in its use among databases is described by Yon Rhee et al (2008) [17].

In other cases, the properties of effectors and other host interaction factors are simply uncharacterized in particular strains or during interactions with particular hosts. In databases where the host taxon is not readily displayed for annotations to terms in the "interaction between organisms" tree or where the host is specified but with an ISS evidence code, users should consider the possibility that the annotation may not be accurate for all source strains and hosts. When involved in generating annotations based on sequence or structural similarity, users should consider avoiding propagation of those most likely to vary based on source and host. Within the ASAP database, annotations likely to be host-dependent are not routinely propagated with the automated annotation systems used to annotate rapidly accumulating sequence data from "next-generation" sequencing technologies, and transitive annotation of effectors is limited to the general term "GO:0052049 interaction with host via protein secreted by type III secretion system".

## Effector repertoire comparison

Although the approaches used in effector characterization and annotation differ between *P. syringae *and *E. coli*, comparison of the assigned terms illustrates how GO can be used to conceptualize the fundamental similarities and differences that exist among different gene products and pathogenic strategies. As previously mentioned, terms such as "GO:0009405 pathogenesis", "GO:0044412 growth or development of symbiont within host", and "GO:0052049 interaction with host via protein secreted by type III secretion system" are broadly applicable to a wide array of effectors in diverse pathosystems. In contrast, other terms are highly specific to effectors from particular pathosystems, revealing fundamental differences in the processes by which Type III effectors influence the bacterial-host interaction. For example, critical stages of adhesion to the host (GO:0044406), are mediated by Type III effectors in *E. coli *and other animal-associated pathogens [18]. In contrast, host adhesion in *P. syringae *is accomplished by fimbriae and exopolysaccharides [19,20] genes which can be further distinguished in the GO with the terms "GO:0043683 type IV pilus biogenesis" and "GO:0045226 extracellular polysaccharide biosynthetic process", respectively. Conversely, "GO:0001907 killing by symbiont of host cells", whether by the natural progression of necrotic disease or by induction of defense-related programmed cell death (captured with the more specific term GO:0052044), is a hallmark of *P. syringae *effector action [21] that is mediated by toxins independent of the T3SS in *E. coli *and other animal pathogens. Examples include cholera toxin deployed by *Vibrio cholera *and pertussis toxin of *Bordetella pertussis*, the secretion properties of which are described with the terms "GO:0052051 interaction with host via protein secreted by type II secretion system" and "GO:0052050 interaction with host via substance secreted by type IV secretion system", respectively. These examples illustrate the value of annotating to multiple terms, where appropriate, so as to maximally capture both shared and divergent properties exhibited by different virulence factors.

Beyond these broad similarities and differences, shared processes and activities at surprisingly specific levels can also be found. For example, selected *Pto *DC3000 and *E. coli *0157:H7 effectors modulate host innate immunity (expressed with GO:0052167 and its child terms), with some specifically demonstrated to negatively regulate host innate immunity induced by pathogen-associated molecular patterns (captured with GO:0052034).

**Figure 2:**
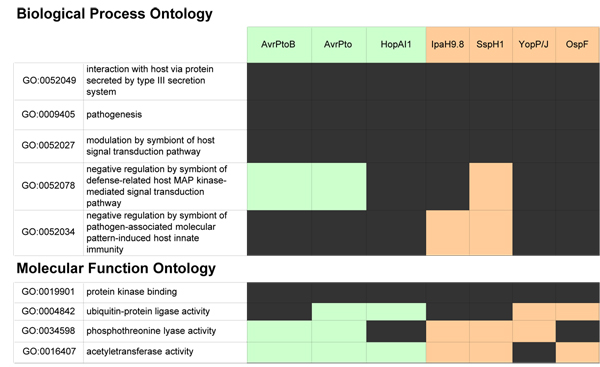
**Comparative Gene Ontology annotation for selected Type III effectors from Pto DC3000 and animal pathogenic genera**. Black indicates the identity of effectors annotated to the specified GO term; green, effectors from plant pathogenic bacteria; orange, effectors from animal pathogenic bacteria.

A further illustration of GO-highlighted similarities is shown for a select group of effectors from multiple pathosystems in the table in Figure 2. In both plant and animal systems, complex signaling pathways mediate the response to detected pathogens, with elements of the intervening signaling pathways representing the most common targets for effector-mediated suppression of the immune response. This property is reflected by annotation of AvrPtoB as well as effectors AvrPto, HopAO1, and HopAI1 (*P. syringae*); IpaH9.8, OspF (*Shigella*); SspH1 (*Salmonella*); and YopP/J (*Yersinia*) to the term "GO:0052027 modulation by symbiont of host signal transduction pathway". For some effectors from both plant and animal pathosystems, the nature of this process has been more intensively characterized, supporting annotation to more specific child terms such as "GO:0052078 negative regulation by symbiont of defense-related host MAP kinase-mediated signal transduction pathway" and "GO:0052034 negative regulation by symbiont of pathogen-associated molecular pattern-induced host innate immunity". In other cases, the effectors in question await in depth evaluation.

Additional information as to how effectors accomplish modulation of host signaling is captured with terms in the Molecular Function Ontology, and once again common strategies are evident among effectors in diverse systems. For AvrPtoB, IpaH9.8, and SspH1, interference with signaling pathways is mediated by ubiquitination of host kinases, activities captured with the molecular function terms "GO:0019901 protein kinase binding" and "GO:0004842 ubiquitin-protein ligase activity" [22-24]. AvrPtoB ubiquitinates host kinases involved in resistance-gene mediated host immunity [24], while SspH1 ubiquitinates PKN1 [22,25], a host kinase integral to innate immune response signaling pathways. The ability of IpaH9.8 to suppress innate immunity also appears linked to ubiquitination – in this case of MAP kinases [22].

Other effectors alter immune signaling pathways using alternative enzymatic activities. These include inactivation of MAP kinase signaling by "GO:0034598 phosphothreonine lyase activity", documented for both OspF [26] and HopAI1 [27], and targeting of MAP kinases by acetyltransferase-activity (GO:0016407) observed for YopP/J [28]. AvrPtoB, in addition to its ubiquitination-dependent suppression of resistance gene-mediated host immunity, suppresses innate immunity through E3-ligase independent targeting of kinase signaling [29]. The specific molecular functions by which this suppression is accomplished have yet to be characterized.

## Putting Gene Ontology to work for you

The *Pto *DC3000 and *E. coli *annotations have been deposited in the GO database where searches can be conducted for GO terms or particular gene products. At that site and through linked resources, users can search for gene products annotated to multiple, user-selected GO terms of interest or analyze microarray data for enrichment of genes annotated to particular terms. The value of the Gene Ontology project is directly proportional to the number and quality of the annotations being contributed, and though intensive efforts have been directed toward annotation of virulence-related gene products in *Pto *DC3000 and *E. coli*, ongoing annotation of these and effectors deployed by a wider range of plant and animal pathogens would greatly enhance the insights that could be gained. Among the steps being taken to facilitate ongoing annotation are proposals that journals request suggested GO terms from manuscript authors akin to requests for keywords. Guidelines are also being developed to aid researchers in identification of appropriate terms for specific protein families. For example, a tutorial on GO term assignment for plant pathogenic effectors is presently available through the Pseudomonas-Plant Interaction website .

For users of GO annotation, it is important to remember that annotations exist within a diffuse network of databases that include the primary, organism-specific databases where annotations are chiefly generated, the GO database itself, and additional sites to which annotation data are downloaded. Significant inconsistencies can and do occur among databases resulting from differences in annotation format, as previously discussed with regard to the "NOT" qualifier, as well as from differences in the frequency of data exchange among databases. In some instances, the differences among databases simply reflect the length of time it takes for changes instituted by the GO Consortium to propagate through the many databases using GO. For example, the dual taxon field pioneered by PAMGO has only recently been added to TIGR-CMR, the database through which *P. syringae *annotations are forwarded to GO. For these reasons, users are encouraged to identify the sources and version numbers of the annotations they are using and include this information in publications making use of these data.

GO annotation represents a vitally important tool for organizing the wealth of biological data that has accompanied the emergence of genomics and high-throughput expression analysis. Through development of terms capturing the interaction between organisms, the PAMGO consortium has added the important domain of interorganismal interactions to the range of processes encompassed by GO, applicable to research on both pathogenic interactions and beneficial symbioses. Creation of the secondary taxon field has additionally provided a means of capturing nuances of interaction observed upon interaction with different hosts. As exemplified by ongoing annotation of effectors in *P. syringae *and *E. coli*, application of these terms to gene products deployed by different organisms interacting with diverse hosts represents a powerful tool for identification of fundamental parallels underlying outwardly dissimilar interactions.

## Competing interests

The authors declare that they have no competing interests.

## References

[B1] Coburn B, Sekirov I, Finlay BB (2007). Type III Secretion Systems and Disease. Clin Microbiol Rev.

[B2] Zhou J-M, Chai J (2008). Plant pathogenic bacterial type III effectors subdue host responses. Current Opinion in Microbiology.

[B3] Marie C, Broughton WJ, Deakin WJ (2001). *Rhizobium *type III secretion systems: legume charmers or alarmers?. Curr Opin Plant Biol.

[B4] Skorpil P, Saad MM, Boukli NM, Kobayashi H, Ares-Orpel F, Broughton WJ, Deakin WJ (2005). NopP, a phosphorylated effector of *Rhizobium *sp. strain NGR234, is a major determinant of nodulation of the tropical legumes *Flemingia congesta *and *Tephrosia vogelii*. Molecular Microbiology.

[B5] Tobe T, Beatson SA, Taniguchi H, Abe H, Bailey CM, Fivian A, Younis R, Matthews S, Marches O, Frankel G (2006). An extensive repertoire of type III secretion effectors in *Escherichia coli *O157 and the role of lambdoid phages in their dissemination. PNAS.

[B6] Lindeberg M, Stavrinides J, Chang JH, Alfano JR, Collmer A, Dangl JL, Greenberg JT, Mansfield JW, Guttman DS (2005). Proposed guidelines for a unified nomenclature and phylogenetic analysis of type III hop effector proteins in the plant pathogen *Pseudomonas syringae*. Mol Plant Microbe Interact.

[B7] Ma W, Dong FF, Stavrinides J, Guttman DS (2006). Type III effector diversification via both pathoadaptation and horizontal transfer in response to a coevolutionary arms race. PLoS Genet.

[B8] Stavrinides J, Ma W, Guttman DS (2006). Terminal Reassortment Drives the Quantum Evolution of Type III Effectors in Bacterial Pathogens. PLoS Pathogens.

[B9] Ashburner M, Ball CA, Blake JA, Botstein D, Butler H, Cherry JM, Davis AP, Dolinski K, Dwight SS, Eppig JT (2000). Gene Ontology: tool for the unification of biology. Nat Genet.

[B10] Buell CR, Joardar V, Lindeberg M, Selengut J, Paulsen IT, Gwinn ML, Dodson RJ, Deboy RT, Durkin AS, Kolonay JF (2003). The complete genome sequence of the *Arabidopsis *and tomato pathogen *Pseudomonas syringae *pv. *tomato *DC3000. Proc Natl Acad Sci USA.

[B11] Lindeberg M, Cartinhour S, Myers CR, Schechter LM, Schneider DJ, Collmer A (2006). Closing the circle on the discovery of genes encoding Hrp regulon members and type III secretion system effectors in the genomes of three model *Pseudomonas syringae *strains. Mol Plant Microbe Interact.

[B12] DeVinney R, Stein M, Reinscheid D, Abe A, Ruschkowski S, Finlay BB (1999). Enterohemorrhagic *Escherichia coli *O157:H7 produces Tir, which is translocated to the host cell membrane but is not tyrosine phosphorylated. Infect Immun.

[B13] Goosney DL, DeVinney R, Finlay BB (2001). Recruitment of cytoskeletal and signaling proteins to enteropathogenic and enterohemorrhagic *Escherichia coli *pedestals. Infect Immun.

[B14] Kenny B, Warawa J (2001). Enteropathogenic Escherichia coli (EPEC) Tir receptor molecule does not undergo full modification when introduced into host cells by EPEC-independent mechanisms. Infect Immun.

[B15] Allen-Vercoe E, Waddell B, Livingstone S, Deans J, DeVinney R (2006). Enteropathogenic *Escherichia coli *Tir translocation and pedestal formation requires membrane cholesterol in the absence of bundle-forming pili. Cell Microbiol.

[B16] Glasner JD, Plunkett G, Anderson BD, Baumler DJ, Biehl BS, Burland V, Cabot EL, Darling AE, Mau B, Neeno-Eckwall EC (2008). Enteropathogen Resource Integration Center (ERIC): bioinformatics support for research on biodefense-relevant enterobacteria. Nucleic Acids Res.

[B17] Yon Rhee S, Wood V, Dolinski K, Draghici S (2008). Use and misuse of the gene ontology annotations. Nat Rev Genet.

[B18] Coburn B, Sekirov I, Finlay BB (2007). Type III secretion systems and disease. Clin Microbiol Rev.

[B19] Ude S, Arnold DL, Moon CD, Timms-Wilson T, Spiers AJ (2006). Biofilm formation and cellulose expression among diverse environmental *Pseudomonas *isolates. Environmental Microbiology.

[B20] Roine E, Raineri DM, Romantschuk M, Wilson M, Nunn DN (1998). Characterization of type IV pilus genes in *Pseudomonas syringae *pv. *tomato *DC3000. Mol Plant-Microbe Interact.

[B21] Greenberg JT (1996). Programmed cell death: A way of life for plants. Proc Natl Acad Sci U S A.

[B22] Rohde JR, Breitkreutz A, Chenal A, Sansonetti PJ, Parsot C (2007). Type III Secretion Effectors of the IpaH Family Are E3 Ubiquitin Ligases. Cell Host & Microbe.

[B23] Abramovitch RB, Janjusevic R, Stebbins CE, Martin GB (2006). Type III effector AvrPtoB requires intrinsic E3 ubiquitin ligase activity to suppress plant cell death and immunity. Proc Natl Acad Sci USA.

[B24] Rosebrock TR, Zeng L, Brady JJ, Abramovitch RB, Xiao F, Martin GB (2007). A bacterial E3 ubiquitin ligase targets a host protein kinase to disrupt plant immunity. Nature.

[B25] Haraga A, Miller SI (2006). A *Salmonella *type III secretion effector interacts with the mammalian serine/threonine protein kinase PKN1. Cellular Microbiology.

[B26] Arbibe L, Kim DW, Batsche E, Pedron T, Mateescu B, Muchardt C, Parsot C, Sansonetti PJ (2007). An injected bacterial effector targets chromatin access for transcription factor NF-[kappa]B to alter transcription of host genes involved in immune responses. Nat Immunol.

[B27] Zhang J, Shao F, Li Y, Cui H, Chen L, Li H, Zou Y, Long C, Lan L, Chai J (2007). A *Pseudomonas syringae *effector inactivates MAPKs to suppress PAMP-induced immunity in plants. Cell Host & Microbe.

[B28] Sweet CR, Conlon J, Golenbock DT, Goguen J, Silverman N (2007). YopJ targets TRAF proteins to inhibit TLR-mediated NF-[kappa]B, MAPK and IRF3 signal transduction. Cellular Microbiology.

[B29] He P, Shan L, Lin N-C, Martin GB, Kemmerling B, Nurnberger T, Sheen J (2006). Specific Bacterial Suppressors of MAMP Signaling Upstream of MAPKKK in *Arabidopsis *Innate Immunity. Cell.

